# Healthcare Professionals’ Perspectives on Agentic Artificial Intelligence in Clinical Work: A Qualitative Study

**DOI:** 10.3390/healthcare14203386

**Published:** 2026-10-11

**Authors:** Stefanie Mache, Lena Klimaschewski, Monika Bernburg, David A. Groneberg, Volker Harth

**Affiliations:** 1 Institute for Occupational and Maritime Medicine (ZfAM), University Medical Center Hamburg-Eppendorf (UKE), 20459 Hamburg, Germany; 2 Institute of Occupational Medicine, Social Medicine and Environmental Medicine, Goethe University Frankfurt, 60590 Frankfurt, Germany

**Keywords:** agentic artificial intelligence, healthcare professionals, qualitative research, digital health, delegated agency, workforce transformation, occupational health, implementation science, clinical workflow

## Abstract

**Background/Objectives:** Artificial intelligence (AI) is increasingly integrated into healthcare, primarily supporting clinical decision-making. Agentic AI may extend these applications by autonomously planning, coordinating, and executing clinical and administrative workflows. However, empirical evidence on how healthcare professionals conceptualise agentic AI and anticipate its implications for work and occupational well-being remains limited. This study explores healthcare professionals’ perceptions and expectations regarding agentic AI and its potential effects on healthcare work. **Methods:** We conducted a qualitative study using semi-structured interviews with 25 healthcare professionals from different clinical backgrounds in Germany. Interviews explored perceptions of agentic AI, focusing on workflow organisation, workload, professional autonomy, accountability, and occupational well-being. Data were analysed using reflexive thematic analysis following Braun and Clarke. **Results:** Five themes were identified: (1) from assisting decisions to organising work; (2) automation redistributes rather than removes work; (3) delegating action without delegating responsibility; (4) implications for working conditions and health- and work-related outcomes; and (5) meaningful human control requires organisational conditions. Participants distinguished between conventional AI-based decision support and more autonomous forms of AI in their accounts, using this contrast to describe potential changes in workflow organisation. Their accounts highlighted practical consequences of delegating workflow-related activities to AI systems, including changes in task structures, monitoring and verification demands, exception management, and the distribution of professional responsibility. Participants anticipated reduced routine administrative workload and improved care coordination, but also potential increases in cognitive and supervisory demands. **Conclusions:** Agentic AI may shift healthcare from AI-assisted decision-making towards AI-mediated workflow execution. Its effects on occupational well-being may therefore be bidirectional, combining workload relief with increased cognitive and supervisory demands. Appropriate governance, clearly defined accountability, meaningful human oversight, workforce training, and regulatory alignment will be essential for responsible implementation.

## 1. Introduction

Healthcare systems worldwide are facing a convergence of demographic, epidemiological, economic and workforce-related challenges that increasingly strain the sustainability of high-quality care. Population ageing and the growing prevalence of chronic conditions and multimorbidity are associated with increasing healthcare needs and utilisation, while persistent workforce shortages and rising healthcare expenditures place additional pressure on health systems and healthcare professionals [1,2,3].

These developments are not only affecting healthcare delivery but are also increasingly shaping the working conditions and occupational well-being of healthcare professionals, with high workloads, occupational stress, and organizational constraints being associated with burnout and lower job satisfaction [4]. At the same time, the growing complexity of clinical decision-making and the increasing burden associated with electronic health records, documentation, and fragmented workflows may further contribute to cognitive and organizational strain in everyday clinical practice [5].

Artificial intelligence (AI) has emerged as one of the most promising technological approaches to address these systemic challenges. In healthcare, AI applications have traditionally focused on narrow, task-specific domains such as medical image analysis, risk prediction, and clinical decision support systems [6]. More recently, the emergence of large language models (LLMs) has expanded the scope of AI applications to include documentation support, information retrieval and patient communication [7]. While these developments have demonstrated potential to reduce certain administrative tasks, most current healthcare AI applications remain human-initiated or human-supervised, with relatively limited autonomy in clinical workflows and continued reliance on clinician review and contextual judgement [8,9,10,11].

A new generation of AI systems, referred to as agentic AI, represents a more fundamental shift. Unlike conventional AI systems, agentic AI is designed to autonomously plan, coordinate and execute multi-step, knowledge-intensive workflows across digital environments [12,13,14]. These systems can integrate structured and unstructured clinical data, interact with electronic health records, access external tools, and adapt their actions based on feedback loops. As such, agentic AI extends beyond decision support towards the delegation of parts of clinical and administrative agency to AI systems [12,15].

This transformation has the potential to significantly reshape healthcare delivery. Anticipated applications include autonomous coordination of appointments and referrals, discharge and follow-up management, chronic disease monitoring, documentation workflows, and inter-organisational care coordination [12,14]. Policy perspectives increasingly suggest that AI could function as an infrastructural technology capable of improving efficiency, reducing administrative burden, and helping healthcare systems respond to workforce shortages [16,17]. Importantly, these developments are closely linked not only to healthcare system performance but also to the working conditions of healthcare professionals, including workload, time pressure, cognitive demands, and burnout [18].

At the same time, the increasing autonomy of AI systems raises important questions regarding their impact on healthcare professionals’ occupational well-being and work-related outcomes. While AI-enabled automation may reduce certain forms of administrative burden, it may simultaneously introduce new demands, including system oversight, verification of AI-generated outputs, coordination of AI-supported workflows, and the management of uncertainty surrounding automated decisions [18,19]. These shifts may influence cognitive workload, work-related strain, professional satisfaction and perceptions of control over clinical work. Thus, the net impact of increasingly autonomous AI on healthcare work remains insufficiently understood and may involve both relieving and burdening mechanisms [18].

Beyond individual-level effects, agentic AI also raises fundamental questions about professional roles, accountability and organisational structures. As AI systems become increasingly capable of initiating and coordinating clinical processes, traditional boundaries between human and technological agency may become increasingly blurred. This raises critical issues related to responsibility, transparency, trust and governance, particularly in high-stakes clinical environments [18]. Furthermore, the redistribution of tasks between healthcare professionals and increasingly autonomous AI systems may reshape patterns of human–AI collaboration and alter the distribution of roles and responsibilities within healthcare teams [20]. However, the longer-term organisational and professional consequences of these changes remain insufficiently understood.

### 1.1. Theoretical Framework

Socio-Technical Systems Theory (STS) served as the overarching conceptual perspective, conceptualizing medical departments as complex socio-technical systems in which technological innovations are embedded within and interact dynamically with social processes, professional roles, organisational structures, workplace culture, and clinical workflows rather than operating in isolation [21]. From this perspective, the effects of AI are not determined solely by its technological capabilities but emerge from the interaction between the technology and the social and organisational context in which it is implemented. Accordingly, the extent to which AI contributes to improved care processes or creates unintended consequences depends substantially on the degree of alignment between technological capabilities, organisational conditions, and the realities of clinical practice [22,23].

The Job Demands–Resources (JD-R) model provided the primary theoretical framework for examining occupational well-being. The model posits that employees’ well-being and work-related outcomes are shaped by the interplay between job demands and job resources [24,25]. Within healthcare, relevant job demands may include high cognitive workload, time pressure, frequent interruptions, emotional demands, complex care coordination, and administrative workload, whereas professional autonomy, social and organisational support, effective teamwork, adequate staffing, and supportive working conditions represent important job resources [26,27]. Against this background, AI was conceptualised as a potentially ambivalent factor that may function either as a job resource by reducing routine and administrative workload, supporting clinical processes, and facilitating care coordination, or as an additional job demand by introducing new responsibilities, monitoring and verification requirements, cognitive demands, and forms of work-related complexity.

To further account for technology-related occupational strain, the study incorporated the concept of technostress [28]. Technostress refers to psychological strain associated with the use and adaptation to information and communication technologies and may arise from technology-induced complexity, information overload, continuous adaptation to changing digital systems, and uncertainty regarding technological reliability [29,30]. Within the context of AI-supported medical care, this perspective provided a framework for examining clinicians’ perceptions of technology-related workload, alert fatigue, documentation burden, the need to monitor and verify AI-generated outputs, and the psychological implications of AI-assisted decision-making [31]. Importantly, technostress complements the JD-R perspective by providing a more technology-specific account of how AI may constitute an additional occupational demand.

Technology acceptance research provided a complementary perspective on healthcare professionals’ willingness to engage with and adopt AI-based technologies. Perceived usefulness, perceived ease of use, trust, and user acceptance have been identified as important factors influencing technology adoption and sustained use [32]. These factors are particularly relevant when introducing increasingly autonomous AI systems into healthcare, as perceived utility, reliability, transparency, and controllability may influence healthcare professionals’ willingness to incorporate such systems into established clinical and organisational workflows.

Human Factors Engineering provided an additional framework for examining the interaction between healthcare professionals, AI systems, and the work environment. This perspective emphasises usability, cognitive workload, situational awareness, communication, and workflow integration as important determinants of safe and effective technology use in complex and safety-critical healthcare environments [22,33]. Together, these perspectives enabled the study to examine not only whether healthcare professionals perceive agentic AI as useful and acceptable, but also how its integration into clinical work may influence cognitive demands, technology-related strain, professional autonomy, and the conditions under which AI may support rather than disrupt healthcare delivery.

### 1.2. Research Gap

Despite growing conceptual and policy interest in agentic AI, empirical evidence on how healthcare professionals perceive increasingly autonomous AI systems remains limited. A recent scoping review identified only a small number of empirical studies specifically examining agentic AI in healthcare, with existing research remaining largely exploratory and focused on technical or clinical outcomes rather than on healthcare professionals’ experiences and working conditions [12]. In contrast, research on AI in healthcare more broadly has predominantly examined clinicians’ interactions with decision-support and other AI-based tools, highlighting factors such as perceived usefulness, trust, explainability, usability, and integration into existing clinical workflows as important determinants of acceptance and effective human–AI collaboration [34,35]. However, it remains unclear whether findings derived primarily from assistive or decision-support applications adequately capture the implications of systems that move beyond recommendation towards autonomous coordination and execution of clinical tasks and workflows.

Importantly, comparatively little is known about how healthcare professionals anticipate the impact of increasingly autonomous AI on working conditions and health- and work-related outcomes, including workload, stress, cognitive burden, job satisfaction, and professional well-being. Emerging evidence from the broader literature on AI in healthcare suggests that such effects may be ambivalent: AI-enabled technologies may reduce aspects of workload while simultaneously introducing new cognitive demands, including the need to monitor and verify AI-generated outputs [18]. Similarly, recent research on AI-enabled workflows has highlighted a potential tension between workload reduction and increased cognitive demands or technostress [36]. Understanding how healthcare professionals perceive these potential changes is therefore important, as such perceptions may influence technology acceptance, implementation processes, and the longer-term sustainability of AI integration within healthcare organisations.

### 1.3. Study Aim

This qualitative study aims to explore healthcare professionals’ perceptions and expectations regarding the emerging role of agentic artificial intelligence (AI) in healthcare delivery. It examines how healthcare professionals anticipate the potential integration of increasingly autonomous AI systems into clinical and administrative work, with particular attention to whether such systems may be perceived as supportive resources that reduce routine workload and facilitate workflow coordination or as potential sources of additional demands, disruption, and uncertainty.

The study specifically explores anticipated implications of agentic AI for workload and working conditions, professional roles and responsibilities, accountability, professional autonomy, human oversight, and the organisation of clinical and administrative workflows. In addition, it examines how healthcare professionals anticipate increasingly autonomous AI systems may affect health- and work-related outcomes, including cognitive workload, stress, professional well-being, and the distribution of work between healthcare professionals and AI systems.

By examining these perceptions and expectations, the study seeks to contribute to a more nuanced understanding of how agentic AI may alter the organisation and distribution of healthcare work and to identify organisational and socio-technical conditions under which its integration may support rather than adversely affect healthcare professionals and working conditions.

The main research question is as follows:

How do healthcare professionals perceive and anticipate the potential implications of agentic artificial intelligence for healthcare work, working conditions, and health- and work-related outcomes?

The following sub-questions are addressed:

1. How do healthcare professionals conceptualise agentic AI and how do they distinguish more autonomous forms of AI from conventional AI-based decision support?
2. To what extent do healthcare professionals perceive agentic AI as a tool for reducing administrative and cognitive workload versus a potential source of additional burden, including new demands such as monitoring, supervision, and coordination of autonomous systems?
3. How do healthcare professionals expect agentic AI to influence clinical decision-making processes and professional autonomy?
4. How do healthcare professionals perceive the implications of delegating operational activities to AI systems for professional accountability and responsibility?
5. How do healthcare professionals anticipate agentic AI will affect working conditions and health- and work-related outcomes, such as workload, stress, cognitive burden, job satisfaction and professional well-being?

## 2. Materials and Methods

### 2.1. Study Design

This study employed an exploratory qualitative design using semi-structured individual interviews to explore healthcare professionals’ experiences, perceptions, and expectations regarding the emerging role of agentic artificial intelligence (AI) in healthcare. The qualitative approach was chosen to capture contextually situated perspectives on how increasingly autonomous AI systems may influence clinical practice, professional roles, working conditions, and health- and work-related outcomes.

The study was informed by a socio-technical perspective, conceptualising healthcare as a complex system in which technological innovations interact with organisational structures, professional practices, clinical workflows, and governance conditions. Given the early stage of agentic AI implementation in healthcare, interviews addressed both participants’ experiences with existing AI-enabled technologies and their expectations regarding the future integration of more autonomous AI systems.

The study was reported in accordance with the Consolidated Criteria for Reporting Qualitative Research (COREQ) [37] (see Supplementary Material S1).

### 2.2. Participant Recruitment and Sampling

Purposive sampling was used to recruit healthcare professionals with diverse professional backgrounds, levels of professional experience, work settings, and familiarity with digital and AI-based technologies. Eligible participants were physicians and nurses working in Germany across inpatient and outpatient healthcare settings, including university hospitals, community hospitals, and ambulatory care organisations. Variation in age, professional experience, healthcare setting, organisational context, and prior exposure to AI was considered during recruitment to capture a range of perspectives.

Participants were recruited through professional networks, institutional contacts, and supplementary snowball sampling. Recruitment requests were sent to 30 clinics and medical departments across different healthcare settings. The requests were forwarded internally by the respective institutions or departments to potentially eligible healthcare professionals. Interested individuals then contacted the research team directly. Consequently, recruitment was based on voluntary self-selection, and the research team did not have access to the number of individuals who received the invitation within each institution or to the number of individuals who considered but declined participation. Therefore, an individual-level response or refusal rate could not be calculated. Potential participants received written information about the study objectives, voluntary participation, confidentiality, data protection, and the handling of interview data. Written informed consent was obtained prior to participation.

Recruitment and data collection proceeded iteratively alongside preliminary analysis. The final sample size was informed by the concept of information power, whereby the amount of information required depends on the specificity of the study aim, the characteristics of the sample, the use of an established interview approach, the quality and depth of the dialogue, and the analytical strategy. In the present study, the research question was focused on healthcare professionals’ experiences and perspectives regarding AI in healthcare, and participants were purposively selected to provide variation in professional background, work setting, experience, and familiarity with AI-based technologies. The semi-structured interviews generated detailed accounts addressing the research questions, and emerging analysis indicated substantial informational depth and richness across the dataset. Recruitment was therefore concluded after 25 interviews when the research team considered the available information sufficient to address the research questions and further interviews were unlikely to substantially extend the range and depth of the emerging interpretative patterns. This judgement was informed by the concept of information power, according to which the required sample size depends on the amount and relevance of information held by the sample in relation to the study aim [38].

### 2.3. Data Collection

Data were collected between November 2025 and February 2026 using semi-structured individual interviews. The interview guide was developed iteratively to address the study aims while allowing participants to describe their perceptions, expectations, and concerns in their own terms. Its development was informed by the empirical and theoretical literature on AI in healthcare, relevant perspectives from implementation science, and emerging conceptual work on agentic and increasingly autonomous AI systems. The five conceptual perspectives underpinning the study—Socio-Technical Systems Theory, the Job Demands–Resources (JD-R) model, technostress, technology acceptance, and Human Factors perspectives—were used as sensitising concepts during interview guide development. Relevant literature and theoretical perspectives were used to identify broad areas of enquiry and to inform the formulation of open-ended questions rather than to establish predefined response categories.

For example, Socio-Technical Systems Theory informed questions concerning the interaction between AI systems, professional roles, tasks, workflows, and organisational structures. The JD-R model informed questions concerning anticipated changes in workload, time pressure, cognitive demands, autonomy, and available resources. Technostress perspectives informed attention to uncertainty, monitoring demands, perceived loss of control, overload, and technology-related strain. Technology acceptance perspectives informed questions concerning perceived usefulness, trust, barriers to adoption, and organisational readiness. Human Factors perspectives informed questions concerning human oversight, error detection, intervention, accountability, and safe human–AI interaction. The frameworks were therefore used to structure broad areas of enquiry rather than to prescribe how participants should respond.

The interview guide covered eight main domains: (1) understanding and conceptualisation of agentic AI; (2) perceived opportunities and potential applications in clinical and organisational workflows; (3) anticipated effects on workload and working conditions; (4) implications for clinical decision-making, professional autonomy, and responsibility; (5) risks related to patient safety, accountability, transparency, and trust; (6) anticipated effects on interprofessional collaboration and patient care; (7) organisational, technical, and governance-related requirements and barriers to implementation; and (8) anticipated effects on health- and work-related outcomes, including stress, cognitive workload, job satisfaction, and professional well-being.

The questions were predominantly open-ended and were supplemented by follow-up prompts where clarification or greater depth was appropriate. Follow-up questions were used to clarify participants’ reasoning, elicit concrete examples, and explore perceived consequences for professional practice. Examples of the interview domains are provided in Supplementary Material S1.

Given the limited empirical evidence and relatively early stage of implementation of agentic AI in healthcare, the interviews addressed both participants’ accounts of existing AI-enabled technologies and their expectations regarding potential future applications of increasingly autonomous systems. This distinction was maintained throughout the interviews. Participants were invited to draw on their current professional experience with AI-based tools where applicable and to reflect separately on potential future applications and implications of agentic AI. Where participants referred to currently used AI-based tools, follow-up questions were used to explore their concrete experiences, including how the technology was used in practice and how it affected their work. Questions concerning agentic AI systems or capabilities not currently used in their professional practice focused on anticipated applications, opportunities, risks, and implications for healthcare work.

Before each interview, participants received a standardised briefing to establish a common working definition of agentic AI. The briefing was intentionally provided before the substantive interview questions to ensure that all participants had a common conceptual starting point. The briefing was used to introduce the topic rather than to prescribe specific responses. The full briefing text is provided in Supplementary Material S2. The briefing was followed by open-ended questions inviting participants to discuss their own understanding, expectations, and perceived implications of agentic AI in healthcare. During the analysis, particular attention was therefore paid to distinguishing characteristics introduced by the briefing from aspects that participants developed further in relation to their own professional contexts, including the delegation of operational activities, monitoring and verification, exception management, professional responsibility, and occupational well-being.

The interview guide was pilot-tested with two healthcare professionals prior to the main data collection. The two pilot interviews were used to assess the clarity, sequencing, comprehensibility, and overall suitability of the questions and were excluded from the final sample of 25 interviews. Based on the pilot interviews, questions were refined with regard to wording, sequencing, and comprehensibility. During the main data collection, the overarching domains and core questions remained stable, while follow-up prompts were refined iteratively in response to emerging issues identified through preliminary analysis. This iterative approach allowed emerging topics to be explored in subsequent interviews without changing the overall scope of the interview schedule.

Interviews were conducted via secure video-conferencing platforms and lasted approximately 30–45 min. All interviews were conducted in German by a female researcher using the semi-structured guide and follow-up questions to explore relevant issues in greater depth. Only the interviewer and the participant were present during the interviews; no other people attended the video interviews. No repeat interviews were conducted. No formal field notes were taken during the interviews. Analytic observations and reflections were documented subsequently in analytic memos as part of the reflexive analytic process. The interviewer had prior methodological experience in qualitative research and expertise in public health, including experience in the design and conduct of qualitative studies. The interviewer had no prior personal or professional relationship with the participants. Interviews were audio-recorded with participants’ informed consent.

Audio recordings were transcribed verbatim in German and checked against the original recordings for accuracy. Transcripts were anonymised prior to analysis by removing or replacing directly identifying information, including names of individuals, institutions, and locations. Transcripts were not returned to participants for comment or correction, and participants were not asked to validate the emerging themes. Interview data were handled in accordance with the study’s data protection and confidentiality procedures.

### 2.4. Data Analysis

Data were analysed using reflexive thematic analysis (RTA) following Braun and Clarke [39,40]. RTA was chosen as a theoretically flexible and interpretative approach for identifying patterns of shared meaning across the dataset while acknowledging the active role of researchers in the development and interpretation of themes. The interviews were conducted and transcribed verbatim in German. Coding and thematic analysis were conducted on the German-language transcripts. Coding was supported by MAXQDA (version 24; VERBI Software, Berlin, Germany).

Given the breadth and richness of the interview material, the dataset covers several interrelated aspects of healthcare professionals’ perceptions and expectations regarding AI in healthcare. The present study focuses specifically on the research questions outlined above. Other aspects of the dataset address distinct research questions and are therefore analysed and reported separately in other publications.

The quotations selected for presentation in the manuscript were subsequently translated from German into English by the first author (see Supplementary Material S4). The translations were checked against the original German transcripts to ensure that the meaning and contextual nuances of participants’ statements were retained. Where necessary, wording was adjusted to preserve the meaning of the original statements while maintaining readability in English.

The five conceptual perspectives were used as sensitising concepts during analysis rather than as predefined coding frameworks. They provided a conceptual background for interpreting patterns in the data and directed attention to issues such as the relationship between technology and work organisation, job demands and resources, technology-related strain, technology acceptance, and human oversight and safety. Initial coding was primarily inductive and data-driven, with codes developed from participants’ accounts rather than assigned according to predefined theoretical constructs. The frameworks therefore informed interpretation without constraining the development of codes or themes.

Initial coding was predominantly semantic, capturing the explicit content and meaning of participants’ statements. Through subsequent comparison and interpretation of codes, the analysis moved towards a more latent level by examining relationships, assumptions, and broader patterns of meaning underlying participants’ accounts. Codes were iteratively developed, merged, refined, and reorganised throughout the analysis. Thus, the final themes were not direct summaries of the topics addressed in the interview guide, but interpretative patterns developed through engagement with the dataset.

Data collection and analysis proceeded iteratively, allowing emerging issues to inform subsequent interviews and sampling. Candidate themes were developed by examining relationships among codes and were repeatedly reviewed against coded extracts and the complete dataset. Themes were refined with regard to internal coherence, distinctiveness, their ability to capture a meaningful pattern across the dataset, and their relevance to the research questions. Particular attention was paid to developing themes around central interpretative concepts rather than reproducing the structure of the interview guide.

Five of the 25 transcripts (20%) were independently reviewed by a second researcher to introduce alternative interpretations and support critical discussion of coding and theme development. Consistent with the principles of reflexive thematic analysis, this process was not intended to establish inter-rater reliability or coding consensus. Instead, differences in interpretation were discussed within the research team to challenge assumptions, explore alternative readings of the data, and refine the developing analytical framework.

The research team comprised researchers with disciplinary expertise in human medicine, psychology, and health sciences, as well as experience in the application and study of artificial intelligence in healthcare. These disciplinary and professional perspectives informed the researchers’ understanding of healthcare work, technology use, and the potential opportunities and challenges associated with AI in healthcare. The researchers therefore entered the analysis with prior knowledge and assumptions regarding clinical practice, human behaviour, organisational contexts, and human–AI interaction. Rather than attempting to bracket these perspectives, the research team reflected on how their disciplinary backgrounds, professional experiences, and theoretical assumptions might shape attention to particular aspects of the data and the interpretation of participants’ accounts.

Epistemologically, the study was positioned within an interpretivist and constructionist perspective, consistent with the reflexive approach to thematic analysis described by Braun and Clarke [39,40]. Participants’ accounts were understood as situated and context-dependent, and the resulting themes were considered interpretative constructions developed through the researchers’ active engagement with the dataset rather than objective representations of an underlying reality. Researcher reflexivity was maintained throughout the analytic process through analytic memos, documentation of key analytic decisions, and regular team discussions. Particular attention was paid to how the researchers’ disciplinary backgrounds, professional experiences, and theoretical assumptions may have influenced the interpretation of participants’ accounts. These reflections were incorporated into ongoing discussions of coding, theme development, and interpretation. The final themes were therefore understood as interpretative constructions developed through sustained engagement with the dataset rather than as objectively discovered categories or descriptive summaries of interview topics [39,40].

### 2.5. Quality Criteria and Trustworthiness

Study quality and trustworthiness were considered in accordance with established qualitative research criteria, including credibility, dependability, confirmability, and transferability [41]. Credibility was supported through the use of a systematically developed semi-structured interview guide, iterative engagement with the data, prolonged examination of transcripts and coded material, and critical discussion within the research team. Independent review of a subset of transcripts by a second researcher provided additional perspectives and supported critical reflection on coding and theme development.

Dependability was enhanced through systematic documentation of methodological and analytical decisions, including changes to the interview guide, sampling considerations, coding development, and theme refinement. An audit trail and analytic memos supported transparency throughout the research process.

Confirmability was addressed through reflexive practice, including documentation and discussion of researchers’ assumptions, disciplinary backgrounds, and potential influences on interpretation. Interpretations were repeatedly examined against the underlying interview data and alternative explanations were considered during theme development.

Transferability was supported by providing detailed descriptions of participant characteristics, professional backgrounds, healthcare settings, organisational contexts, and the analytic procedures. This enables readers to assess the applicability of the findings to other healthcare contexts.

### 2.6. Ethical Considerations

This study was conducted with human participants in accordance with the principles of the Declaration of Helsinki.

All participants received written and verbal information about the study and provided informed consent prior to participation. Participation was voluntary, and participants could withdraw at any time without providing a reason. Data were anonymised and stored securely in accordance with data protection regulations.

## 3. Results

### 3.1. Sociodemographic Characteristics of Participants

A total of 25 healthcare professionals participated in the study. The sample comprised 11 physicians and 14 nurses. Participants represented a range of clinical perspectives and were recruited from different healthcare settings.

The sample showed variation in age, professional experience, and organisational context. Participants were drawn from both inpatient and outpatient care environments, including university hospitals, non-university hospitals and ambulatory care settings.

Detailed sociodemographic and professional characteristics of the participants are presented in Table 1.

### 3.2. Overview of Qualitative Findings

Table 2 summarises the five themes developed from the qualitative interviews.

The themes describe how healthcare professionals responded to and elaborated on the concept of agentic AI introduced in the standardised interview briefing: (1) From assisting decisions to organising work; (2) Automation redistributes rather than removes work; (3) Delegating action without delegating responsibility; (4) Implications for working conditions and health- and work-related outcomes; and (5) Meaningful human control requires organisational conditions.

In their accounts, participants themselves distinguished between conventional AI-based decision support and more autonomous forms of AI. This distinction was not explicitly introduced in the interview briefing but emerged as participants described what greater AI autonomy could mean for healthcare work. Their accounts extended the initial description of agentic AI by addressing changes in task structures, the distribution of work and responsibility, supervisory and verification demands, professional autonomy, and occupational well-being. These accounts informed the development and interpretation of the five themes.

### 3.3. From Assisting Decisions to Organising Work

Across both professional groups, participants themselves articulated a distinction between conventional AI-based decision support and more autonomous forms of AI. This distinction was not explicitly introduced in the interview briefing but emerged in participants’ accounts of what greater AI autonomy could mean for healthcare work. Physicians and nurses similarly described a potential shift from consulting AI-based tools towards delegating parts of a workflow to systems that could initiate and coordinate multiple consecutive activities. Their accounts, however, extended beyond the characteristics presented in the briefing by focusing on what increased AI autonomy could mean for the organisation of healthcare work. In particular, participants discussed the transfer of operational activities to AI systems, the coordination of tasks across multiple workflow stages, and the resulting need for human monitoring and oversight.

One physician described this anticipated development as follows:

*“This will go far beyond mere support (…). Behind the scenes, it would organize the entire workflow.”*

(P07, physician)

A nurse similarly described the potential shift from using AI as a tool towards AI taking a more active role in managing processes:

*“If the system could do the coordination of the next steps automatically, I think that would take a lot of the organisational work off us. (…) but I would still want to know what it has done and where things have been changed, especially if it affects the patient.”*

(P01, nurse)

Another physician emphasised the same conceptual shift from using AI as a tool towards AI taking an active role in managing parts of a workflow:

*“It kind of feels like it’s not just a tool you ask anymore, but something that actually starts managing parts of the system itself.”*

(P12, physician)

Thus, both professional groups described agentic AI primarily in terms of a transition from individual decision support towards more extensive delegation of workflow-related activities. Their accounts extended the initial concept of autonomous action by considering how AI could affect the organisation and distribution of operational work. Rather than focusing solely on whether AI systems could initiate or coordinate actions, participants discussed the consequences of delegating activities that are currently performed or coordinated by healthcare professionals.

Participants associated this potential shift with what can be characterised as delegated agency: selected clinical or administrative activities could be transferred from healthcare professionals to AI systems. Frequently mentioned areas of potential application included appointment scheduling, referral coordination, documentation, communication, and discharge management. Participants thus extended the initial concept of autonomous task execution by considering how AI could operate across several stages of care delivery.

Importantly, participants did not describe autonomy as uniformly desirable or undesirable. Instead, both physicians and nurses differentiated between types of tasks and contexts. Administrative activities such as scheduling, documentation, or coordination were generally discussed as areas in which greater delegation might be feasible, whereas clinical activities involving uncertainty, risk, or professional judgement were associated with greater concerns regarding autonomous execution. The acceptability of AI autonomy was therefore discussed as context-dependent and related to the potential consequences of an AI-initiated action.

Participants also discussed questions of human monitoring and responsibility in relation to delegation. These aspects are examined in greater detail in Section 3.4 and Section 3.5. At this stage, participants primarily described delegation as a transformation in how work could be organised rather than as a complete replacement of professional activity.

### 3.4. Automation Redistributes Rather than Removes Work

Participants’ accounts concerning agentic AI were predominantly anticipatory in this domain. Both physicians and nurses anticipated that agentic AI could reduce routine administrative work, particularly in relation to documentation, coordination, scheduling, and communication. Such applications were perceived as having the potential to release time for direct patient care and other activities requiring human interaction or professional judgement.

A physician emphasised the potential time-saving effect of automation:

*“If it really takes over the paperwork and coordination, that will save a huge amount of time.”*

(P03, physician)

Nurses similarly anticipated time savings but more explicitly highlighted the possibility that automated work would still require human verification:

*“The work doesn’t really go away. It just shifts. You’re not doing the tasks yourself anymore, but more like keeping an eye on what the system is doing.”*

(P18, nurse)

*“If the documentation is done automatically, we’d obviously have more time for the patients. But I’d still want to check what the system actually entered.”*

(P02, nurse)

Thus, both professional groups anticipated that automation could reduce direct administrative work, while the nurses’ accounts presented here placed particular emphasis on the additional checking and oversight that might accompany such automation. This illustrates that perceived workload reduction was not necessarily understood as an overall reduction in work, but rather as a redistribution of tasks between direct execution and supervisory activities.

Several participants described an additional layer of work associated with monitoring, checking, correcting, and managing AI-supported processes. We interpreted this pattern as a form of ‘second-order workload‘, referring to work that arises not from the primary task itself but from the need to supervise and manage AI-supported task execution. This included monitoring AI-generated actions, evaluating whether automated processes were appropriate, identifying errors or deviations, and intervening when systems encountered situations outside their expected operating conditions.

A physician similarly emphasised that expected time savings could be accompanied by additional responsibility for verification:

*“Theoretically, you do gain some time because the AI program takes over some of the routine tasks. But at the same time, you then also have to check everything the system does (…). So, it’s not really less work (…) you’re just doing a different kind of work.”*

(P11, physician)

Taken together, participants across both professional groups differentiated between task-related workload and oversight-related workload. Although the former could potentially be reduced through automation, the latter could increase as AI systems became more autonomous. Several participants were concerned that continuous monitoring requirements could create cognitive fragmentation, particularly when AI-driven processes operated simultaneously across multiple workflows.

The anticipated consequences depended strongly on the reliability and predictability of the AI system. Participants suggested that poorly integrated or insufficiently reliable systems could generate additional work through exception handling and repeated verification, potentially offsetting expected efficiency gains.

### 3.5. Delegating Action Without Delegating Responsibility

Across both professional groups, participants described accountability as remaining with healthcare professionals despite increasing AI autonomy. Physicians and nurses shared the expectation that increasing AI autonomy would not necessarily be accompanied by a corresponding transfer of professional responsibility. At the same time, their accounts placed somewhat different emphases on this issue. Physicians more explicitly framed the issue in terms of professional accountability and responsibility for adverse outcomes, whereas nurses more directly emphasised the practical responsibility of recognising and responding to inappropriate AI-supported actions in patient care.

A physician described the persistence of professional responsibility despite increasing AI autonomy:

*“If something goes wrong, in the end it’s still me who is responsible. Even if the system made the decision, or whatever, I’m the one who has to deal with the patient. So I can’t really say, ‘The AI did it, not me.’ I mean, the responsibility is still there.”*

(P09, physician)

Nurses similarly emphasised that healthcare professionals would remain responsible for detecting and addressing problematic AI-supported actions:

*“The system makes the decision, yes, but (…) we still have to check whether something is wrong. And then, I mean, we’re the ones who have to intervene.”*

(P04, nurse)

*“If the AI does something wrong, well, the patient isn’t going to talk to the AI about it. They’re going to come to us, obviously. And then we have to deal with it, even if it wasn’t actually us who made the decision.”*

(P24, nurse)

Thus, the two professional groups converged on the perceived persistence of human responsibility, although nurses’ accounts more explicitly connected this responsibility to the practical realities of patient care and intervention.

The perceived responsibility gap was also expressed in more general terms by a physician:

*“We can delegate the task to the system, but in the end we are still responsible for what happens. If something goes wrong, I don’t think you can simply say that the AI made the decision. Someone still has to take responsibility for it.”*

(P15, physician)

These accounts highlighted a potential asymmetry between delegated operational action and retained professional responsibility. Participants did not necessarily expect responsibility to be transferred together with the delegated task. Instead, they anticipated situations in which AI systems could perform or initiate activities while healthcare professionals remained responsible for monitoring these activities and responding to their consequences.

This perceived asymmetry was closely connected to concerns about professional autonomy and control. Some participants anticipated that increasing reliance on autonomous systems could gradually shift decision-making authority away from healthcare professionals and constrain their ability to determine how clinical work is organised.

*“If the system starts deciding more and more, you slowly lose control over your own work.”*

(P09, physician)

Other participants interpreted this development less as a loss of autonomy and more as a transformation of professional roles. Rather than performing routine tasks directly, healthcare professionals might increasingly assume supervisory functions involving system monitoring, exception management, validation, and governance. This perspective implied the emergence of new competencies related to evaluating AI behaviour, recognising system limitations, and determining when human intervention was required.

Participants’ accounts suggested that the perceived consequences of agentic AI for professional autonomy depended not only on the degree of technical autonomy but also on how decision authority, accountability, and opportunities for human intervention were organisationally defined. Thus, the implications of delegated agency appeared to depend not simply on whether tasks were transferred to AI systems, but on how responsibility, control, and intervention rights were distributed within the surrounding socio-technical work system.

### 3.6. Implications for Working Conditions and Health- and Work-Related Outcomes

Participants anticipated both beneficial and adverse consequences of agentic AI for working conditions and health- and work-related outcomes. Across both professional groups, potential reductions in administrative and repetitive tasks were associated with expectations of lower time pressure and greater availability for patient-facing activities. Nurses’ accounts particularly emphasised the potential value of spending more time with patients, whereas physicians also highlighted the possibility that reducing routine work could increase the meaningfulness of professional activities.

A nurse described the potential benefit for patient care:

*“If it really works the way they say it will, then maybe we would finally have more time for the patients. Because right now there’s just so much of the other stuff, documentation and all that, that takes up the time.”*

(P05, nurse)

A physician similarly associated the reduction in routine work with greater meaningfulness:

*“If it removes routine work, it could actually make the job more meaningful again.”*

(P08, physician)

Another nurse linked reduced documentation and coordination directly to the quality of the working day:

*“If I spend less time documenting and coordinating, I could spend more time with patients. That would make a real difference to my working day.”*

(P23, nurse)

At the same time, participants across professional groups described potential sources of additional occupational strain. These included continuous monitoring of AI-driven processes, uncertainty regarding system behaviour, responsibility for detecting errors, and dependence on automated systems.

*“It might reduce paperwork, but it increases the mental load of supervising everything.”*

(P14, physician)

Nurses similarly anticipated that potential efficiency gains might be offset by continuous monitoring demands:

*“I could see it going either way, really. If it takes away a lot of the routine work, then maybe we would have less stress and less burnout. But if you’re constantly thinking, okay, what is the system doing now, do I need to check this, did it get that right (…) then that could also be exhausting. So, yeah, maybe it’s just a different kind of overload.”*

(P13, nurse)

*“It might save time, but if I have to constantly check what the system is doing, I don’t know whether I would actually feel less stressed.”*

(P25, nurse)

These accounts indicated convergence across professional groups regarding the potentially dual nature of agentic AI: automation could reduce some sources of workload while simultaneously creating new cognitive and supervisory demands. Nurses’ accounts presented here placed particular emphasis on the immediate consequences for patient-facing time and perceived stress, whereas physicians also described changes in the meaningfulness and mental demands of professional work.

A recurring concern involved continuous attentional demands, particularly where AI systems operated in the background while healthcare professionals remained responsible for monitoring their actions.

*“You are never fully ‘done’ anymore, because the system keeps running in the background. Even if you’ve finished your own work, you still have to think about what the AI system is doing and whether you need to intervene. So, I think that could make it difficult to really switch off after the work is done.”*

(P16, nurse)

These accounts suggested that automation might change not only the amount of work but also its temporal and cognitive characteristics. Participants anticipated a potential shift from periods of task completion towards ongoing supervisory attention, with possible implications for perceived control, recovery from work, and cognitive workload.

Participants therefore described agentic AI as having a potentially dual impact on health- and work-related outcomes: it could function as a resource by reducing repetitive and administrative demands, while simultaneously creating new demands related to supervision, verification, uncertainty, and responsibility.

### 3.7. Meaningful Human Control Requires Organisational Conditions

When discussing the conditions for future implementation of agentic AI, participants from both professional groups emphasised trust, transparency, and organisational readiness. Trust was perceived to depend not only on system performance but also on the ability to understand, monitor, and retrospectively assess AI-driven actions.

A physician emphasised the importance of understanding AI behaviour for trust:

*“I need to understand why it acts, otherwise I cannot trust it in clinical care.”*

(P06, physician)

A nurse similarly stressed the need to understand system behaviour and retain the ability to challenge or override it:

*“(…) I need to know why the system is doing something and what I can do if I disagree.”*

(P18, nurse)

Thus, physicians and nurses converged on the importance of transparency and comprehensibility, while the nurse account additionally highlighted the practical importance of retaining the ability to disagree with or intervene in AI-supported actions.

Participants described transparency, explainability, traceability, and auditability as important requirements, particularly when AI systems were able to initiate actions without immediate human input. Concerns about automation bias were also raised, with participants describing the possibility that repeated exposure to apparently reliable AI recommendations or actions could reduce critical scrutiny.

*“The danger is that people stop questioning the system because it usually works.”*

(P16, nurse)

At the organisational level, participants emphasised the need for clearly defined governance structures specifying which activities could be performed autonomously, which required human approval, and under which circumstances human intervention had to be triggered.

*“There must be clear boundaries between what the system can do and what must always stay human.”*

(P10, physician)

Training and digital competence were also perceived as important prerequisites for future implementation. Participants highlighted the need not only to understand how to use AI systems but also to recognise their limitations, interpret their outputs, identify potentially inappropriate actions, and intervene when necessary.

*“Without training and involvement, this will either be rejected or blindly trusted.”*

(P21, nurse)

Participants further emphasised the importance of involving healthcare professionals in the design and implementation of AI-supported workflows. Participatory implementation was perceived as a means of ensuring that system functionality corresponded to actual clinical requirements and that new responsibilities were clearly defined.

Across the interviews, participants linked the conditions for meaningful human control not solely to the technical maturity or performance of AI systems but also to organisational readiness, clearly defined roles and responsibilities, appropriate governance mechanisms, and the integration of AI into existing professional and clinical workflows.

## 4. Discussion

### 4.1. Healthcare Professionals’ Perceptions and Expectations

This qualitative study explored healthcare professionals’ perceptions and expectations regarding the emerging role of agentic artificial intelligence (AI) in healthcare delivery. In their accounts, participants distinguished between conventional AI-based decision support and more autonomous forms of AI, describing the latter in terms of systems that could initiate, coordinate, and execute activities within clinical and administrative workflows rather than primarily providing information, predictions, or recommendations in response to human input.

Three interconnected findings are particularly relevant. First, participants described potential changes in how work is organised as selected elements of task execution and workflow coordination could be transferred to AI systems while human professionals retain varying degrees of oversight and responsibility. We characterise this pattern analytically as delegated agency. Second, participants anticipated distribution rather than an unequivocal reduction in workload, with routine administrative tasks potentially being reduced or transferred while new supervisory, verification, and exception-management activities may emerge. Third, participants described agentic AI as having potentially dual implications for occupational well-being. It could reduce time-consuming and repetitive work while simultaneously introducing new cognitive demands, uncertainty, and responsibility.

These findings extend the existing literature on AI in healthcare, which has largely focused on decision support, prediction, diagnosis, documentation, and human–AI collaboration. Recent reviews indicate that successful AI implementation depends strongly on workflow integration, usability, trust, transparency, training, and organisational context rather than on technical performance alone [12,14,42,43]. Importantly, although research on AI agents and agentic AI is expanding rapidly, the evidence base remains relatively immature, with many studies still limited to experimental, benchmark, or controlled evaluation settings and comparatively little evidence from real-world clinical deployment [12,14,44].

These findings suggest that agentic AI may warrant consideration beyond the established paradigm of clinical decision support. In the present accounts, participants associated greater AI autonomy with changes in how healthcare activities could be initiated, coordinated, and executed, and with a redistribution of selected tasks and responsibilities between human professionals and AI systems. Rather than implying a uniform transition away from human decision-making, this perspective highlights how increasing AI autonomy may reshape the organisation of healthcare work and the conditions under which professional oversight and responsibility are exercised.

### 4.2. Agentic AI as a Socio-Technical Transformation

The concept of delegated agency provides a useful analytical lens for understanding the implications of agentic AI in healthcare. Previous literature has conceptualised AI use in terms of delegating selected decision-making and task-related functions to algorithmic systems, including elements of clinical decision-making and healthcare administration [45]. In the present study, participants’ accounts further illustrated how greater AI autonomy may be associated with changes in the organisation and distribution of healthcare work. Participants described a potential shift from systems that primarily support human cognition towards systems that may initiate actions and coordinate sequences of tasks. This distinction is consequential because autonomous action may alter the relationship between technology, work tasks, and professional responsibility.

This interpretation is consistent with socio-technical approaches to healthcare AI, which conceptualise AI not as an isolated technological intervention but as one component of a broader sociotechnical work system. Salwei and Carayon emphasise that the successful application of AI requires consideration of the entire work system and clinical workflow, including interactions among people, tasks, technologies, and organisational structures [45]. Empirical research further suggests that digital technologies can reconfigure professional practices, coordination processes, temporal demands, and accountability within healthcare organisations [46,47].

The present findings extend existing models of human–AI collaboration [48]. Recent reviews of healthcare AI have predominantly examined collaboration in terms of task allocation, decision support, and diagnostic interpretation [12,20,48]. Participants’ accounts suggest that greater AI autonomy introduces an additional dimension because the system may not only contribute to a decision but also initiate downstream actions and coordinate multiple steps within a workflow. This raises questions not only about whether AI improves decision accuracy, but also about how agency, control, and responsibility are distributed across human and AI actors.

At the same time, the findings suggest that the relationship between AI-supported work and greater delegation should not be understood as a simple binary. AI can function as an interactive support tool while healthcare professionals remain actively involved in defining tasks, providing contextual information, evaluating outputs, and redirecting the interaction. Conversely, greater autonomy may involve professionals defining an overarching goal or set of constraints while the AI system determines, sequences, or coordinates intermediate actions with less direct human input. The degree of delegation therefore depends not only on the technical capabilities of the AI system but also on how the system is embedded in the task and workflow.

This perspective is also consistent with research on iterative human–AI interaction. Recent empirical work by Dahò and Caci illustrates how different expert-developed prompting approaches can influence AI-generated clinical rehabilitation plans, while also demonstrating the continued importance of professional evaluation of the resulting outputs [49]. Their findings provide an example of AI-assisted clinical work in which the professional shapes the task and the resulting output through the formulation of prompts, while the AI contributes to the generation of a proposed clinical plan. This type of interaction can therefore be situated between conventional decision support and more extensive delegation of clinical or organisational tasks. It illustrates that human agency may remain substantial even when AI contributes significantly to the content and structure of the resulting output.

Accordingly, we conceptualise delegated agency as a continuum involving both the scope of activities transferred to an AI system and the intensity of ongoing human involvement. At lower levels of delegation, professionals may formulate specific tasks, provide instructions, evaluate individual outputs, and repeatedly redirect the interaction. At higher levels, professionals may define an overarching goal or set of constraints while the AI system determines, sequences, or coordinates intermediate actions with less direct human input. Importantly, greater technical autonomy does not necessarily correspond to lower human agency: a highly capable system may still operate under intensive human supervision, whereas a more limited system may perform a substantial task with comparatively little intervention.

In this sense, delegated agency is an analytical characterisation of a pattern identified in the present study rather than a concept explicitly used by participants. Delegation does not necessarily imply disengagement or the relinquishment of professional agency. Rather, agency may be redistributed across different stages of a workflow. Healthcare professionals may retain agency through goal setting, task definition, contextualisation, output evaluation, approval, correction, and intervention, while AI systems assume greater responsibility for executing or coordinating intermediate activities. The resulting configuration is therefore better characterised as a redistribution of agency than as a straightforward transfer of agency from humans to machines.

Importantly, participants did not describe autonomy as uniformly desirable or undesirable. Rather, the acceptability of autonomous action was perceived as dependent on the task, context, and potential consequences of AI action. Administrative activities such as scheduling, documentation, or coordination were generally considered more amenable to delegation, whereas activities involving greater uncertainty, risk, or professional judgement raised stronger concerns. This task- and consequence-dependent understanding of autonomy provides an important empirical qualification of the broader concept of delegated agency.

The findings also indicate that delegation of operational activities does not necessarily imply a corresponding transfer of professional responsibility. Even where AI systems were envisioned as performing or coordinating tasks, participants anticipated that healthcare professionals would retain responsibility for monitoring, evaluating, and intervening in AI-supported processes. This creates a distinction between delegated action and retained accountability: responsibility may remain with professionals even when operational agency is partially transferred to AI systems.

This does not imply that professional autonomy will necessarily decline. Some participants interpreted the emergence of supervisory roles as a transformation rather than a loss of professional autonomy. Professional autonomy may increasingly involve determining when AI should act, evaluating whether its actions are appropriate, intervening in exceptional situations, and governing the boundaries of autonomous operation. Thus, implementation strategies should avoid equating human involvement simply with retaining a nominal “human in the loop.” Instead, human involvement needs to provide meaningful authority, competence, and opportunities for intervention [19].

### 4.3. Workload Transformation

The anticipated reduction in administrative burden was one of the most prominent perceived benefits of agentic AI. Participants expected potential applications in documentation, scheduling, referral coordination, communication, and discharge management to reduce repetitive tasks and release time for patient-facing activities. These expectations are consistent with emerging empirical evidence from less autonomous AI applications. Studies of ambient AI documentation technologies, for example, have reported reductions in documentation burden and aspects of cognitive and temporal demands among clinicians [50,51,52].

However, the findings of the present study suggest that such benefits cannot be understood solely in terms of the amount of work performed. Participants repeatedly described a potential shift from task execution towards supervision and verification. Rather than simply eliminating tasks, increasingly autonomous systems may redistribute work by transferring some activities from direct execution to monitoring, reviewing, and verifying AI-generated outputs. This distinction is particularly relevant to the occupational implications of agentic AI. Recent evidence indicates that the cognitive effects of AI in healthcare vary substantially according to the type of AI application. A 2026 systematic review and meta-analysis found evidence of reduced cognitive workload for some ambient documentation applications, whereas findings for diagnostic AI and clinical decision-support systems were mixed. The authors also identified verification burden as an important area requiring further investigation [18].

From a Job Demands–Resources perspective, agentic AI may therefore function simultaneously as a job resource and a job demand [53]. Recent research applying the JD-R framework to AI-driven healthcare similarly conceptualizes AI-related changes in physicians’ work as potential job demands while identifying AI-related competencies as job resources that may buffer their negative effects [54]. The net effect is therefore likely to depend not only on the capabilities of the technology but also on how it is integrated into work processes, how much supervision it requires, and whether sufficient organisational resources are available to support that supervision.

This interpretation is also consistent with research on technostress in healthcare. Healthcare professionals already experience technology-related stressors, particularly techno-overload and techno-uncertainty, which are associated with the increasing demands and unpredictability of digital health technologies [55]. In the context of AI-powered healthcare systems, recent research has further identified techno-unpredictability as a potential stressor, reflecting difficulties arising from the unpredictable outcomes of AI-based systems [56]. Increasingly autonomous AI systems may therefore amplify existing technostress mechanisms when professionals are required to continuously monitor, interpret or verify AI-generated outputs. At the same time, the effects of AI on workload are not necessarily negative. Recent evidence suggests that well-integrated AI applications, particularly ambient AI documentation, can reduce aspects of cognitive workload and work exhaustion, whereas diagnostic AI and clinical decision-support systems may produce more mixed effects [18]. Accordingly, the success of agentic AI should not be evaluated exclusively through conventional efficiency indicators such as time saved, tasks automated, or throughput. Rather, implementation evaluations should also consider how AI redistributes cognitive demands and supervisory responsibilities, including measures of cognitive workload, monitoring and verification demands, professional autonomy, and burnout alongside efficiency outcomes [18]. This broader perspective is particularly important for agentic AI, where reductions in visible workload may coexist with less visible forms of cognitive and supervisory burden.

### 4.4. Trust, Transparency and Meaningful Human Oversight

Trust was identified by participants as a prerequisite for the adoption of agentic AI, but the findings suggest that trust is closely linked to transparency, reliability, predictability, and controllability. This is consistent with recent systematic evidence showing that healthcare professionals’ trust in AI-based clinical decision-support systems is influenced by system transparency, training, usability, clinical reliability, human-centred design, and the ability to retain control over the technology [43]. Reviews of medical AI transparency similarly emphasise that the ability to understand, interpret and verify AI system behaviour is central to safe clinical integration [57,58].

The importance of these factors may be amplified in agentic systems. In a conventional decision-support scenario, a clinician can decide whether to act on an AI recommendation. In an agentic workflow, by contrast, the system may initiate actions, use tools and coordinate multiple steps across a clinical workflow. Consequently, meaningful oversight may require clinicians to understand not only the content of an AI recommendation, but also the system’s current state, actions already taken, intended next steps, and conditions for escalation, intervention, or termination [12,59].

This creates a broader requirement for operational transparency. Explainability of individual model outputs may not be sufficient when AI systems operate across multi-step workflows. Users may require visibility into system state, actions taken, decision thresholds, escalation rules, and audit trails [12,59]. This distinction is particularly important in high-stakes clinical environments.

Participants also raised concerns regarding automation bias. This concern is consistent with evidence showing that excessive reliance on automated recommendations can reduce critical scrutiny and may impair human–AI decision-making when clinicians defer to incorrect AI outputs [57]. Recent research therefore emphasises the importance of calibrated or “appropriate” trust rather than maximising trust in AI systems.

Conversely, insufficient or poorly calibrated trust may contribute to reluctance, rejection, or workarounds that limit the potential benefits of AI-supported decision-making [43].

Meaningful human oversight therefore requires more than the formal presence of a human decision-maker. The EU AI Act explicitly requires human oversight of high-risk AI systems to be effective and proportionate to the risks, level of autonomy, and context of use. It further requires deployers to assign human oversight to persons with the necessary competence, training, authority and support [60].

This regulatory principle closely reflects the concerns raised by participants. However, the findings suggest that human oversight should also be understood as a work-design and organisational issue: oversight that is technically possible but operationally too demanding may become ineffective in practice [19].

### 4.5. Accountability and Professional Responsibility

The tension between delegated agency and retained professional responsibility represents one of the most consequential findings of this study. Participants anticipated situations in which AI systems may initiate or execute actions while healthcare professionals remain responsible for the resulting outcomes. This perceived mismatch can be conceptualised as an accountability gap, in which operational agency and normative responsibility are not necessarily aligned.

This issue extends existing debates surrounding clinical AI. As AI becomes increasingly embedded in healthcare decision-making, responsibility cannot be attributed exclusively to the technical system. Rather, accountability is distributed across multiple actors involved in the development, deployment and use of AI, including developers, healthcare professionals and organisations. Recent research on medical AI ethics similarly identifies transparency, accountability, human autonomy, and organisational responsibility as persistent governance challenges [61,62].

Agentic AI potentially intensifies this challenge because its role extends beyond generating informational outputs to initiating and coordinating actions. As systems become increasingly capable of acting with limited human intervention, explicit responsibility structures become essential before deployment. Healthcare professionals cannot reasonably be expected to assume full responsibility for actions they cannot adequately observe, understand, interrupt, or reconstruct. Meaningful human oversight therefore requires not merely human involvement, but sufficient capacity to understand system behaviour and intervene when necessary [19].

This suggests that accountability should be understood as a property of the broader sociotechnical system rather than solely as an individual professional obligation. Responsibility structures should specify accountability for system selection, configuration, validation, monitoring, incident management, and individual clinical decisions. Importantly, responsibility for the AI system itself should be distinguished from responsibility for decisions made in individual patient encounters [63].

The findings therefore support a shift from the generic concept of a “human in the loop” towards a more explicit model of human authority, defined system boundaries and distributed accountability. This distinction is particularly relevant for agentic AI, where increasing system autonomy may otherwise create a gap between formal human responsibility and the practical capacity to supervise and control system behaviour [19].

### 4.6. Implications for Occupational Health and Workforce Sustainability

The findings are consistent with emerging empirical evidence suggesting that AI can have heterogeneous effects on healthcare professionals’ workload and well-being. Recent studies of ambient AI documentation technologies indicate reductions in documentation burden and aspects of cognitive workload, while effects on burnout, professional fulfilment, and broader well-being appear more heterogeneous and may depend on the specific AI application, implementation context, and duration of use [18,50,52,64]. A recent systematic review and meta-analysis similarly found that the cognitive impact of AI varies substantially according to the specific application. Ambient AI documentation was associated with reductions in cognitive workload and burnout, but the certainty of evidence was moderate for cognitive workload and low for burnout. The authors therefore conclude that the overall impact of clinical AI on healthcare professionals’ workload and well-being remains uncertain and context-dependent [18].

From an occupational health perspective, this suggests that the relevant question is not simply whether AI “reduces workload.” Instead, implementation should consider which demands are removed, which new demands are introduced and who bears them. Agentic AI may reduce clerical demands while increasing supervisory demands; it may reduce interruptions associated with manual coordination while introducing new attentional requirements and it may increase professional capacity while simultaneously creating greater dependence on technological infrastructure.

This finding can be interpreted directly through the Job Demands–Resources model. AI-enabled automation may operate as a job resource when it reduces repetitive demands, increases available time or improves access to information. However, the same technology may constitute a job demand when it increases monitoring requirements, uncertainty, cognitive workload or responsibility. The occupational consequences of agentic AI are therefore likely to depend on the balance between these mechanisms rather than on automation per se.

Accordingly, agentic AI should be conceptualised not merely as a productivity technology but as a work-design intervention with potential occupational health consequences. Future implementation studies should therefore evaluate not only productivity and clinical outcomes but also validated measures of workload, burnout, cognitive demands, professional autonomy, job satisfaction, and recovery from work.

### 4.7. Governance and Policy Implications

The findings have important implications for AI governance, particularly in the European context. The EU AI Act establishes specific requirements for high-risk AI systems, including risk management, documentation, monitoring and effective human oversight [60]. However, it would be inappropriate to assume that all agentic AI applications in healthcare are automatically classified as high-risk. Classification depends on the system’s intended purpose and its specific use under the regulatory framework. Nevertheless, agentic systems used for certain clinical or safety-relevant purposes may fall within high-risk categories, making the principles of human oversight, risk management, and lifecycle monitoring highly relevant.

Participants’ emphasis on clear autonomy boundaries, traceability, human intervention, and organisational responsibility is therefore consistent with broader international governance recommendations. The World Health Organization emphasises the need for transparency, accountability, stakeholder engagement, human oversight, and continuous assessment in the development and deployment of AI for health [61]. A recent review similarly suggests that trustworthy healthcare AI requires attention to workflow fit, organisational readiness, human oversight, and post-deployment monitoring, rather than relying exclusively on pre-deployment technical validation [65].

The findings further suggest that governance should be operationalised at the organisational level. Policies should specify which tasks may be delegated to AI, which actions require human confirmation, what constitutes an exception requiring escalation, and how AI-related incidents are reported and reviewed. Such governance mechanisms should be integrated into everyday clinical workflows rather than treated as separate compliance procedures.

Participatory implementation is another important implication. Study participants emphasised training, digital competence, and involvement in system design. This is consistent with socio-technical and human-centred approaches that emphasise the involvement of clinical end users throughout the design, implementation, and evaluation of AI systems, thereby helping to ensure that technologies are aligned with users’ needs and actual clinical work practices [66]. For agentic AI, participation may be particularly important because clinicians are not merely users of a tool but may become supervisors and governors of an autonomous workflow component [44].

### 4.8. Strengths and Limitations

This study has several strengths. It addresses an emerging area for which empirical evidence remains limited. Recent reviews have identified agentic AI as an emerging field in healthcare while highlighting limited clinical evidence and substantial gaps in evaluation and validation [12,14]. The inclusion of participants from different professional groups and contexts allowed the analysis to capture perspectives extending beyond a single occupational or technological setting. This supported a socio-technical interpretation of agentic AI that considers changes in tasks, workflows, professional roles, responsibility, and organisational conditions. The study also explicitly examined occupational and organisational consequences, areas that have received comparatively less attention than technical performance and clinical accuracy in healthcare AI research.

Several limitations should nevertheless be considered.

Because agentic AI remains at an early stage of clinical implementation, many participant accounts were anticipatory rather than based on sustained real-world experience. Expectations may differ from experiences following actual deployment, particularly as users become familiar with a technology, encounter unexpected system behaviour, and adapt their work practices. Consequently, findings concerning workload, professional autonomy, accountability, and occupational well-being should be interpreted as participants’ perceptions and expectations rather than as evidence of the actual effects of deployed agentic AI systems.

The study was conducted within the German healthcare context. Regulatory structures, professional roles, digital infrastructures, organisational conditions, and patterns of technology adoption differ across healthcare systems. These contextual characteristics may have shaped participants’ expectations regarding the feasibility, acceptability, and governance of agentic AI. The findings should therefore be understood as contextually situated rather than universally transferable.

Recruitment through professional networks and institutional contacts, including snowball sampling, may have introduced self-selection bias. Participants with a particular interest in digitalisation, AI, or healthcare innovation may have been more likely to participate or recommend other potential participants. This may have increased the visibility of both opportunities and concerns associated with AI compared with perspectives among healthcare professionals with less engagement with digital technologies. The findings should therefore not be interpreted as representing the views of healthcare professionals as a whole. At the same time, the inclusion of participants with different professional backgrounds provided variation in the perspectives represented in the dataset.

The study relied on self-reported perceptions, experiences, and expectations and did not objectively measure workload, cognitive load, burnout, patient outcomes, or the performance of AI systems. The findings therefore identify perceived mechanisms and potential consequences rather than establishing causal effects. In particular, statements concerning stress, burnout, job satisfaction, or occupational well-being describe participants’ anticipated or perceived implications and should not be interpreted as evidence of measured changes in these outcomes.

The standardised briefing provided at the beginning of each interview may have influenced how participants conceptualised and discussed agentic AI. The briefing necessarily introduced specific characteristics, including autonomous goal pursuit, context-dependent decision-making, interaction with digital systems and the initiation or coordination of actions. Findings concerning these characteristics should therefore not be interpreted as entirely independent, participant-generated definitions of agentic AI. Instead, the analysis examined how participants elaborated on the briefing and applied the concept to their own professional contexts, particularly with regard to workflow delegation, task- and context-dependent autonomy, monitoring, verification, exception management, professional responsibility, and occupational well-being. Although this approach provided a common conceptual starting point across interviews, a framing effect cannot be excluded.

The distinction between participants’ experiences with currently deployed AI-supported tools and their expectations regarding future agentic AI systems represents an additional limitation. Some participants were able to draw on direct experience with existing AI-enabled applications, whereas many statements concerning agentic AI were necessarily anticipatory because comparable systems are not yet routinely implemented in their professional settings. Although the analysis distinguished experience-based accounts from anticipatory accounts where this was identifiable, participants’ expectations about future systems may have been shaped by their existing experiences with other forms of AI as well as by public and professional discourse about emerging technologies. Accordingly, anticipatory findings should not be interpreted as evidence of actual effects following implementation.

The interviews lasted approximately 30–45 min and covered eight broad domains. The semi-structured design allowed participants to elaborate on issues they considered particularly relevant, but the available interview time necessarily limited the depth with which every domain could be explored in every interview. This may have resulted in some topics receiving more detailed discussion than others and may have limited the development of particularly nuanced accounts in less extensively discussed areas. The final themes should therefore be understood as patterns developed across the dataset rather than as comprehensive accounts of every domain for every participant.

The qualitative sample was necessarily limited in size and diversity. In keeping with the logic of reflexive thematic analysis, the purpose of the sample was not statistical representation or to establish whether themes would be reproduced in a larger population. Rather, the analysis sought to develop theoretically and empirically grounded interpretations of patterns of meaning within the dataset. The composition of the sample nevertheless affects the range of perspectives available for analysis. Participants from professional groups, organisational settings, or healthcare contexts not represented in the study may have conceptualised agentic AI differently or emphasised other concerns. Future research could therefore extend the present findings by examining these interpretations in other professional, organisational, and healthcare contexts rather than by treating replication of the themes as a criterion of validity.

Rapid developments in agentic AI may change both technological capabilities and healthcare professionals’ expectations over relatively short periods. The findings should therefore be regarded as temporally situated. As agentic systems become more widely implemented, longitudinal and empirical research will be needed to examine how expectations identified in this study compare with experiences following deployment, including changes in workload, professional roles, accountability, and occupational well-being.

### 4.9. Research Implications

The findings identify several priorities for future research. The concept of delegated agency warrants further theoretical development and empirical investigation. Future studies should examine how agency is distributed between humans and AI across different levels of autonomy and different types of clinical and administrative tasks. Particular attention should be given to the relationship between autonomy, professional judgement, accountability and human intervention.

Longitudinal and implementation-focused research is needed to distinguish anticipated from experienced effects. Studies conducted before, during and after implementation could examine how AI changes task allocation, workflow coordination, supervisory activity, and professional perceptions over time.

The occupational consequences of agentic AI should be assessed using validated measures. Future mixed-methods studies could combine qualitative investigation with measures of cognitive workload, burnout, job satisfaction, professional autonomy, technostress, interruptions, and recovery from work. Such studies would help determine whether reductions in administrative workload are offset by increases in supervisory or verification demands.

Research should investigate the organisational conditions under which human oversight remains effective. This includes examining appropriate staffing levels for supervision, escalation mechanisms, workload thresholds, interface design, and the distribution of responsibility across professionals, organisations, and AI developers.

Future studies should adopt a multi-stakeholder perspective involving healthcare professionals, patients, managers, developers, regulators, and other relevant actors. This would allow questions of trust, accountability, acceptable autonomy, and governance to be examined across the healthcare ecosystem rather than from the perspective of individual users alone.

### 4.10. Practical Implications

The findings suggest that successful implementation of agentic AI will depend not only on technical performance but also on organisational design and workforce adaptation.

Healthcare organisations should anticipate a potential shift from direct task execution towards supervision, validation, and exception management. Workflow redesign should therefore ensure that human oversight is both technically possible and practically manageable. Excessive monitoring demands may otherwise undermine the workload reductions expected from automation.

Organisations should establish explicit governance structures defining the boundaries of AI autonomy, human intervention requirements, escalation pathways, and responsibility allocation. These mechanisms should be operationalised within everyday clinical workflows rather than treated solely as regulatory documentation.

Training should extend beyond technical system use. Healthcare professionals should be equipped to understand system capabilities and limitations, recognise failure modes, interpret AI behaviour, assess uncertainty, and intervene appropriately. Training should therefore encompass both AI literacy and supervisory competence.

Implementation should involve healthcare professionals throughout system selection, workflow design, configuration, testing, and evaluation. Participatory approaches may improve workflow fit, usability, trust, and acceptance while reducing the risk of unintended work intensification.

Organisations should evaluate agentic AI using a multidimensional set of outcomes. Efficiency and productivity measures should be complemented by indicators of workload, cognitive demands, professional autonomy, staff well-being, patient safety, and care quality.

Policymakers and healthcare organisations should consider agentic AI as a potential health-system and workforce intervention, rather than solely as a digital technology. Its potential to increase capacity in settings affected by workforce shortages will only translate into sustainable benefits if technological efficiency is accompanied by appropriate work design, governance, and protection of healthcare professionals’ occupational well-being.

## 5. Conclusions

Agentic artificial intelligence may represent a significant development in healthcare technology, with potential implications extending beyond established forms of AI-based decision support. In this study, healthcare professionals described how greater AI autonomy could enable the initiation, coordination, and partial execution of clinical and administrative workflow activities. We use the concept of delegated agency as an analytical characterisation of this pattern to describe how selected operational activities may be transferred to AI systems while human professionals retain varying degrees of oversight and responsibility. Participants’ accounts indicated that the acceptability of AI autonomy depends on the specific task, context, and potential consequences of AI action.

Participants perceived substantial potential for reducing routine administrative work and improving workflow coordination. At the same time, they anticipated new demands associated with monitoring, verification, uncertainty, accountability, and professional control. Importantly, participants described a potential asymmetry between delegated action and retained responsibility: operational activities may be transferred to AI systems while healthcare professionals remain responsible for monitoring these processes and responding to their consequences. Agentic AI may therefore transform rather than simply reduce healthcare work by shifting activities from direct task execution towards supervision, verification, and exception management.

The successful integration of agentic AI will require more than technological maturity. It will depend on meaningful human oversight, clearly defined accountability structures, workflow-compatible system design, appropriate training, participatory implementation, and systematic monitoring of occupational and clinical outcomes. In particular, implementation should consider whether efficiency gains are accompanied by increased cognitive or supervisory demands for healthcare professionals.

Ultimately, the challenge is not simply whether agentic AI can perform increasingly autonomous tasks, but how autonomous action can be embedded within healthcare work in ways that preserve patient safety, meaningful professional agency, and sustainable working conditions. The findings suggest that the implications of agentic AI will depend substantially on how autonomy, responsibility, and opportunities for human intervention are defined within the broader socio-technical work system.

## Figures and Tables

**Table 1:** Sociodemographic and professional characteristics of study participants (*n* = 25).

Characteristic	Category	*n*	%
Professional group	Physicians	11	44.0
	Nurses	14	56.0
Gender	Female	13	52.0
	Male	12	48.0
Age group	30–39 years	5	20.0
	40–49 years	10	40.0
	50–59 years	8	32.0
	≥60 years	2	8.0
Professional experience	<5 years	2	8.0
	5–10 years	5	20.0
	>10 years	18	72.0
Organisational context	University hospital	9	36.0
	Community/non-university hospital	8	32.0
	Ambulatory care organisation	8	32.0

Note: Percentages are based on the total sample (*n* = 25) and may not sum to exactly 100% due to rounding.

**Table 2:** Main themes and subthemes from qualitative interviews on agentic AI in healthcare.

Main Theme	Subthemes
1. From assisting decisions to organising work	- Implications of moving from reactive decision support towards proactive AI-mediated workflows - AI as workflow coordinator across organisational boundaries - Integration of clinical and administrative processes - Blurring boundaries between tool and actor
2. Automation redistributes rather than removes work	- Reduction of administrative burden (documentation, scheduling, coordination) - Emergence of supervisory and monitoring tasks - Second-order workload (system oversight and verification) - Cognitive fragmentation and changing task structures
3. Delegating action without delegating responsibility	- Responsibility allocation in autonomous AI actions - Liability concerns in case of errors or adverse events - Perceived responsibility gap between action and accountability - Concerns about loss or transformation of professional autonomy
4. Implications for working conditions and health- and work-related outcomes	- Potential reduction of time pressure and administrative strain - Increased cognitive load due to supervision requirements - Effects on stress, burnout risk, and emotional burden - Impact on job satisfaction and perceived meaningfulness of work - Uncertainty about net effects on occupational well-being
5. Meaningful human control requires organisational conditions	- Need for explainability and auditability of AI decisions - Risks of automation bias and overreliance - Requirements for human oversight and escalation pathways - Training needs and digital literacy gaps - Importance of participatory design and clinician involvement in implementation

## Data Availability

The datasets analyzed during the current study are not publicly available due to German national data protection regulations but are available from the corresponding author on reasonable request.

## Supplementary Materials

The following supporting information can be downloaded at: https://www.mdpi.com/article/10.3390/healthcare14203386/s1. Supplementary Material S1: Briefing; Supplementary Material S2: Development of the interview guide; Supplement Material S3: COREQ (COnsolidated criteria for REporting Qualitative research) Checklist; Supplementary Material S4: Selected interview quotations on the perceived impact of AI-enabled systems on work and professional responsibilities.

## Abbreviations

AI	Artificial Intelligence
COREQ	Consolidated Criteria for Reporting Qualitative Research
