# Supplementary material for: Healthcare Professionals’ Perspectives on Agentic Artificial Intelligence in Clinical Work: A Qualitative Study
Source: Healthcare (Basel). 2026 Oct 11;14(20):3386. doi: 10.3390/healthcare14203386 (PMC13704506; doi:10.3390/healthcare14203386)
Supplement: Supplementary file 1 [file healthcare-14-03386-s001.zip › healthcare-4572195-supplementary.pdf]

Supplement Material S1: Briefing

The full briefing was as follows:
"Before the interview, we would like to briefly explain what we mean by the term "agentic artificial intelligence" or "agentic AI".

Agentic AI refers to AI systems that can pursue defined goals by independently determining and executing multiple, context-dependent steps, interacting with digital systems and initiating or coordinating actions with a degree of autonomy, while remaining subject to human and organisational oversight.

In the following interview, we are interested in your own understanding and expectations regarding such systems in healthcare. There are currently different possible forms and applications of agentic AI, and there is no single established way in which these systems must be implemented. We are therefore interested in how you perceive their potential role in healthcare and how you think they might affect clinical and administrative work."

The briefing was followed by open-ended questions inviting participants to discuss their own understanding, expectations, and perceived implications of agentic AI in healthcare.

Supplementary Material S2

Table: Development of the interview guide

**Interview domain**	**Main focus / research question**	**Example**
Understanding of agentic AI	How do healthcare professionals conceptualise increasingly autonomous AI?	"How do you understand the term agentic AI in healthcare?"
Workflows and task delegation	How might AI change tasks and workflows?	"Where could you imagine such systems taking over or coordinating tasks?"
Workload and working conditions	What changes in demands and resources are anticipated?	"What could change in your workload or working conditions?"
Decision-making and responsibility	How might autonomy affect professional responsibility?	"Who should be responsible if an AI system initiates an inappropriate action?"
Trust and safety	What risks and safeguards are anticipated?	"What would you need to trust an AI system in this context?"
Collaboration and patient care	How might AI affect interprofessional work and care?	"How could AI change collaboration within your team?"
Implementation and governance	What organisational/technical conditions are required?	"What would need to be in place before such a system could be implemented?"
Well-being and health	What effects on stress, cognitive workload and well-being are anticipated?	"How might such systems affect stress or professional well-being?"

Supplement Material S3: COREQ (COnsolidated criteria for REporting Qualitative research) Checklist

A checklist of items that should be included in reports of qualitative research. You must report the page number in your manuscript where you consider each of the items listed in this checklist. If you have not included this information, either revise your manuscript accordingly before submitting or note N/A.

Topic	Item No.	Guide Questions/Description	Reported on Page No.
**Domain 1: Research team and reflexivity**			
Personal characteristics			
Interviewer/facilitator	1	Which author/s conducted the interview or focus group?	8
Credentials	2	What were the researcher's credentials? E.g. PhD, MD	8
Occupation	3	What was their occupation at the time of the study?	8
Gender	4	Was the researcher male or female?	8
Experience and training	5	What experience or training did the researcher have?	8
Relationship with participants			
Relationship established	6	Was a relationship established prior to study commencement?	7
Participant knowledge of the interviewer	7	What did the participants know about the researcher? e.g. personal goals, reasons for doing the research	8
Interviewer characteristics	8	What characteristics were reported about the inter viewer/facilitator? e.g. Bias, assumptions, reasons and interests in the research topic	8
**Domain 2: Study design**			
Theoretical framework			
Methodological orientation and Theory	9	What methodological orientation was stated to underpin the study? e.g. grounded theory, discourse analysis, ethnography, phenomenology, content analysis	7
Participant selection			
Sampling	10	How were participants selected? e.g. purposive, convenience, consecutive, snowball	6ff
Method of approach	11	How were participants approached? e.g. face-to-face, telephone, mail, email	6ff
Sample size	12	How many participants were in the study?	6ff.
Non-participation	13	How many people refused to participate or dropped out? Reasons?	6ff.
Setting			
Setting of data collection	14	Where was the data collected? e.g. home, clinic, workplace	6 ff.
Presence of non-participants	15	Was anyone else present besides the participants and researchers?	6ff.
Description of sample	16	What are the important characteristics of the sample? e.g. demographic data, date	10-11
Data collection			
Interview guide	17	Were questions, prompts, guides provided by the authors? Was it pilot tested?	8
Repeat interviews	18	Were repeat inter views carried out? If yes, how many?	8
Audio/visual recording	19	Did the research use audio or visual recording to collect the data?	8-9
Field notes	20	Were field notes made during and/or after the inter view or focus group?	9
Duration	21	What was the duration of the inter views or focus group?	8
Data saturation	22	Was data saturation discussed?	8ff.
Transcripts returned	23	Were transcripts returned to participants for comment and/or	7

Topic	Item No.	Guide Questions/Description	Reported on Page No.
		correction?	
**Domain 3: analysis and findings**			
Data analysis			
Number of data coders	24	How many data coders coded the data?	8ff.
Description of the coding tree	25	Did authors provide a description of the coding tree?	9
Derivation of themes	26	Were themes identified in advance or derived from the data?	9
Software	27	What software, if applicable, was used to manage the data?	9
Participant checking	28	Did participants provide feedback on the findings?	9
Reporting			
Quotations presented	29	Were participant quotations presented to illustrate the themes/findings? Was each quotation identified? e.g. participant number	13ff.
Data and findings consistent	30	Was there consistency between the data presented and the findings?	13 ff
Clarity of major themes	31	Were major themes clearly presented in the findings?	13ff
Clarity of minor themes	32	Is there a description of diverse cases or discussion of minor themes?	13ff.

Developed from: Tong A, Sainsbury P, Craig J. Consolidated criteria for reporting qualitative research (COREQ): a 32-item checklist for interviews and focus groups. *International Journal for Quality in Health Care*. 2007. Volume 19, Number 6: pp. 349 – 357

Once you have completed this checklist, please save a copy and upload it as part of your submission. DO NOT include this checklist as part of the main manuscript document. It must be uploaded as a separate file.

Supplementary Material S4. Selected interview quotations on the perceived impact of AI-enabled systems on work and professional responsibilities

Section	Theme	Participant	Professional group	English quotation presented in manuscript	German quotation
3.3	From assisting decisions to organising work	P07	Physician	"This will go far beyond mere support (…). Behind the scenes, it would organize the entire workflow."	„Das wird weit über eine bloße Unterstützung hinausgehen (…). Im Hintergrund würde es den gesamten Arbeitsablauf organisieren."
3.3	From assisting decisions to organising work	P01	Nurse	"If the system could do the coordination of the next steps automatically, I think that would take a lot of the organisational work off us. (…) but I would still want to know what it has done and where things have been changed, especially if it affects the patient."	„Wenn das System die Koordination der nächsten Schritte automatisch übernehmen könnte, würde uns das meiner Meinung nach einen großen Teil der organisatorischen Arbeit abnehmen. (…) Aber ich würde trotzdem wissen wollen, was es getan hat und wo etwas verändert wurde, insbesondere wenn dies den Patienten betrifft."
3.3	From assisting decisions to organising work	P12	Physician	"It kind of feels like it's not just a tool you ask anymore, but something that actually	„Es fühlt sich irgendwie so an, als wäre das nicht mehr nur ein Werkzeug, das

Section	Theme	Participant	Professional group	English quotation presented in manuscript	German quotation
				starts managing parts of the system itself.”	man fragt, sondern etwas, das dann schon selbst Teile des Systems steuert.“
3.4	Automation redistributes rather than removes work	P03	Physician	“If it really takes over the paperwork and coordination, that will save a huge amount of time.”	„Wenn es tatsächlich die Dokumentation und Koordination übernimmt, wird das enorm viel Zeit sparen.“
3.4	Automation redistributes rather than removes work	P18	Nurse	“The work doesn’t really go away. It just shifts. You’re not doing the tasks yourself anymore, but more like keeping an eye on what the system is doing.”	„Die Arbeit verschwindet ja nicht. Sie verlagert sich einfach. Man macht die Aufgaben dann nicht mehr selbst, sondern muss eher schauen, was das System macht.“
3.4	Automation redistributes rather than removes work	P02	Nurse	“If the documentation is done automatically, we’d obviously have more time for the patients. But I’d still want to check what the system actually entered.”	„Wenn die Dokumentation automatisch gemacht wird, hätten wir natürlich mehr Zeit für die Patienten. Aber ich würde trotzdem noch mal schauen wollen, was das Programm tatsächlich eingetragen hat.“

Section	Theme	Participant	Professional group	English quotation presented in manuscript	German quotation
3.4	Second-order workload	P11	Physician	“Theoretically, you do gain some time because the AI program takes over some of the routine tasks. But at the same time, you then also have to check everything the system does (…). So, it’s not really less work (…) you’re just doing a different kind of work.”	„Man gewinnt theoretisch schon Zeit, weil das KI-Programm einige Routineaufgaben übernimmt. Aber gleichzeitig muss man dann auch alles überprüfen, was das System macht (…). Also wirklich weniger Arbeit ist es nicht (…) man macht dann einfach eine andere Art von Arbeit.“
3.5	Accountability and retained responsibility	P09	Physician	“If something goes wrong, in the end it’s still me who is responsible. Even if the system made the decision, or whatever, I’m the one who has to deal with the patient. So, I can’t really say, ‘The AI did it, not me.’ I mean, the responsibility is still there.”	„Wenn etwas schiefgeht, bin am Ende immer noch ich verantwortlich. Selbst wenn das System die Entscheidung getroffen hat oder was auch immer, bin ich derjenige, der sich mit dem Patienten auseinandersetzen muss. Ich kann also nicht wirklich sagen: ‚Die KI war es, nicht ich.‘ Ich meine, die Verantwortung bleibt bestehen.“

Section	Theme	Participant	Professional group	English quotation presented in manuscript	German quotation
3.5	Accountability and retained responsibility	P04	Nurse	“The system makes the decision, yes, but(…) we still have to check whether something is wrong. And then, I mean, we’re the ones who have to intervene.”	„Das System trifft die Entscheidung, ja, aber (…) wir müssen trotzdem überprüfen, ob etwas nicht stimmt. Und dann, ich meine, wir sind diejenigen, die eingreifen müssen.“
3.5	Accountability and retained responsibility	P24	Nurse	“If the AI does something wrong, well, the patient isn’t going to talk to the AI about it. They’re going to come to us, obviously. And then we have to deal with it, even if it wasn’t actually us who made the decision.”	„Wenn die KI etwas falsch macht, wird der Patient natürlich nicht mit der KI darüber sprechen. Er wird zu uns kommen. Und dann müssen wir uns darum kümmern, auch wenn wir die Entscheidung eigentlich gar nicht selbst getroffen haben.“
3.5	Accountability and retained responsibility	P15	Physician	“We can delegate the task to the system, but in the end we are still responsible for what happens. If something goes wrong, I don't think you can simply say that the AI made	„Wir können die Aufgabe an das System delegieren, aber am Ende sind wir trotzdem dafür verantwortlich, was passiert. Wenn etwas schiefgeht, kann man meiner Meinung nach nicht einfach sagen, dass die KI die

Section	Theme	Participant	Professional group	English quotation presented in manuscript	German quotation
				the decision. Someone still has to take responsibility for it.”	Entscheidung getroffen hat. Jemand muss weiterhin die Verantwortung dafür übernehmen.“
3.5	Professional autonomy and control	P09	Physician	“If the system starts deciding more and more, you slowly lose control over your own work.”	„Wenn das System zunehmend mehr Entscheidungen trifft, verliert man langsam die Kontrolle über die eigene Arbeit.“
3.6	Working conditions and patient-facing time	P05	Nurse	“If it really works the way they say it will, then maybe we would finally have more time for the patients. Because right now there’s just so much of the other stuff, documentation and all that, that takes up the time.”	„Wenn es wirklich so funktioniert, wie sie sagen, dann hätten wir vielleicht endlich mehr Zeit für die Patienten. Denn im Moment gibt es einfach so viel von dem anderen Zeug, Dokumentation und all das, was die Zeit in Anspruch nimmt.“
3.6	Meaningfulness of work	P08	Physician	“If it removes routine work, it could actually make the job more meaningful again.”	„Wenn es Routinearbeiten abnimmt, könnte die Arbeit tatsächlich wieder sinnvoller werden.“

Section	Theme	Participant	Professional group	English quotation presented in manuscript	German quotation
3.6	Working conditions and patient-facing time	P23	Nurse	"If I spend less time documenting and coordinating, I could spend more time with patients. That would make a real difference to my working day."	„Wenn ich weniger Zeit mit Dokumentation und Koordination verbringe, könnte ich mehr Zeit mit den Patienten verbringen. Das würde für meinen Arbeitsalltag einen echten Unterschied machen."
3.6	Occupational strain	P14	Physician	"It might reduce paperwork, but it increases the mental load of supervising everything."	„Es könnte den Dokumentationsaufwand reduzieren, aber es erhöht die mentale Belastung, alles überwachen zu müssen."
3.6	Stress and digital overload	P13	Nurse	"I could see it going either way, really. If it takes away a lot of the routine work, then maybe we would have less stress and less burnout. But if you're constantly thinking, okay, what is the system doing now, do I need to check this, did it get that right (…) then that could also	„Ich könnte mir wirklich vorstellen, dass es in beide Richtungen geht. Wenn es viel von der Routinearbeit abnimmt, hätten wir vielleicht weniger Stress und weniger Burnout. Aber wenn man ständig darüber nachdenkt: Okay, was macht das System gerade, muss ich das überprüfen, hat es das richtig gemacht (…), dann könnte das auch anstrengend sein. Also ja, vielleicht ist

Section	Theme	Participant	Professional group	English quotation presented in manuscript	German quotation
				be exhausting. So, yeah, maybe it’s just a different kind of overload.”	es einfach eine andere Art von Überlastung.“
3.6	Stress and digital overload	P25	Nurse	“It might save time, but if I have to constantly check what the system is doing, I don't know whether I would actually feel less stressed.”	„Es könnte Zeit sparen, aber wenn ich ständig überprüfen muss, was das System macht, weiß ich nicht, ob ich mich dadurch tatsächlich weniger gestresst fühlen würde.“
3.6	Continuous attentional demands	P16	Nurse	“You are never fully ‘done’ anymore, because the system keeps running in the background. Even if you’ve finished your own work, you still have to think about what the AI system is doing and whether you need to intervene. So, I think that could make it difficult to	„Man ist nie mehr vollständig ‚fertig‘, weil das System im Hintergrund weiterläuft. Selbst wenn man seine eigene Arbeit beendet hat, muss man weiterhin darüber nachdenken, was die KI macht und ob man eingreifen muss. Ich glaube daher, dass es dadurch schwierig werden könnte, nach der Arbeit wirklich abzuschalten.“

Section	Theme	Participant	Professional group	English quotation presented in manuscript	German quotation
				really switch off after the work is done.”	
3.7	Trust and transparency	P06	Physician	“I need to understand why it acts, otherwise I cannot trust it in clinical care.”	„Ich muss verstehen, warum es so handelt, sonst kann ich ihm in der klinischen Versorgung nicht vertrauen.“
3.7	Trust and human control	P18	Nurse	“(…) I need to know why the system is doing something and what I can do if I disagree.”	„(…) Ich muss wissen, warum das System etwas tut und was ich tun kann, wenn ich damit nicht einverstanden bin.“
3.7	Automation bias	P16	Nurse	“The danger is that people stop questioning the system because it usually works.”	„Die Gefahr besteht darin, dass die Menschen aufhören, das System zu hinterfragen, weil es normalerweise funktioniert.“
3.7	Governance and human boundaries	P10	Physician	“There must be clear boundaries between what the system can do and what must always stay human.”	„Es muss klare Grenzen zwischen dem geben, was das System tun kann, und dem, was immer dem Menschen vorbehalten bleiben muss.“

Section	Theme	Participant	Professional group	English quotation presented in manuscript	German quotation
3.7	Training and implementation	P21	Nurse	"Without training and involvement, this will either be rejected or blindly trusted."	„Ohne Schulung und Einbindung wird dies entweder abgelehnt oder blind vertraut."
